# RASSF1A and the Taxol Response in Ovarian Cancer

**DOI:** 10.1155/2012/263267

**Published:** 2012-04-03

**Authors:** Susannah Kassler, Howard Donninger, Michael J. Birrer, Geoffrey J. Clark

**Affiliations:** ^1^J.G. Brown Cancer Center, University of Louisville, 417 CTR Building, 505 S. Hancock Street, Louisville, KY 40202, USA; ^2^Massachusetts General Hospital Cancer Center, Massachusetts General Hospital, Harvard Medical School, Boston, MA 02114, USA

## Abstract

The RASSF1A tumor suppressor gene is frequently inactivated by promoter methylation in human tumors. The RASSF1A protein forms an endogenous complex with tubulin and promotes the stabilization of microtubules. Loss of RASSF1A expression sensitizes cells to microtubule destabilizing stimuli. We have observed a strong correlation between the loss of RASSF1A expression and the development of Taxol resistance in primary ovarian cancer samples. Thus, we sought to determine if RASSF1A levels could dictate the response to Taxol and whether an epigenetic therapy approach might be able to reverse the Taxol resistant phenotype of RASSF1A negative ovarian tumor cells. We found that knocking down RASSF1A expression in an ovarian cancer cell line inhibited Taxol-mediated apoptosis and promoted cell survival during Taxol treatment. Moreover, using a combination of small molecule inhibitors of DNA Methyl Transferase enzymes, we were able restore RASSF1A expression and Taxol sensitivity. This identifies a role for RASSF1A in modulating the tumor response to Taxol and provides proof of principal for the use of epigenetic therapy to overcome Taxol resistance.

## 1. Introduction

RASSF1A is a poorly understood tumor suppressor that can modulate the cell cycle, tubulin dynamics and apoptosis [1–3]. It is subjected to epigenetic inactivation at high frequency in a broad range of human tumors, including approximately 50% of ovarian tumors [1, 4, 5]. Overexpression of RASSF1A promotes hyperstabilization of microtubules reminiscent of Taxol [6, 7], and previous investigations have shown that loss of RASSF1A sensitizes cells to microtubule destabilizing drugs such as nocodazole [7]. Thus, RASSF1A appears to play an important role in modulating microtubule stabilization. This implies that the RASSF1A levels in a tumor cell may impact how the cell responds to Taxol treatment. The development of resistance to Taxol remains a serious problem in the treatment of ovarian cancer.

 The most frequent mechanism by which RASSF1A is inactivated in tumors is by hypermethylation promoter leading to transcriptional silencing [1, 4, 5]. Thus, the gene remains intact, just dormant. Over recent years, a series of small molecules have been identified that can inhibit the DNA methylation system and restore expression of genes that have suffered aberrant promoter methylation [8]. This has given rise to the concept of epigenetic therapy, whereby a tumor would be treated with drugs to restore the expression and function of RASSF1A or some other epigenetically inactivated target. If RASSF1A plays a key role in the response to Taxol, epigenetic therapy could be potentially serve as an approach to overcome the resistance.

In an attempt to address the issue of RASSF1A expression and Taxol resistance, we measured the expression levels of RASSF1A in a series of primary ovarian tumor samples that were characterized for resistance or sensitivity to Taxol. The results showed a very strong correlation between the reduced relative expression of RASSF1A and Taxol resistance in primary ovarian cancer. We then used an shRNA-based approach to generate a matched pair of ovarian tumor cell lines that were positive or negative for RASSF1A expression. In this system, loss of RASSF1A impaired the ability of Taxol to promote microtubule polymerization and rendered the cells resistant to the growth inhibitory effects of Taxol. Using an epigenetic therapy approach, we found that reactivating RASSF1A expression in a RASSF1A-negative ovarian tumor cell line enhanced the sensitivity of the cells to Taxol. Thus we confirm the hypothesis that RASSF1A plays a role in the cellular response to Taxol and provide proof of principal for the use of epigenetic therapy as strategy to address the problem of Taxol resistance ovarian cancer.

## 2. Materials and Methods

### 2.1. Tissue Culture

A547 and UCI-107 cells were grown in DMEM/10% FBS. Cells were transfected with shRNA constructs described previously [9] using lipofectamine 2000 (Invitrogen, Carlsbad, CA, USA) using the manufacturers protocol and selected in 1 *μ*g/mL puromycin. Cells were treated with Taxol (Sigma, St. Louis, MI, USA) at the described doses for 48 hours prior to assay. Cell numbers were measured by trypsinization and counting in a haemocytometer. Cells were treated with Zebularine [10] and/or RG108 [11] dissolved in DMSO for 48 hours prior to assay. *t*-tests were used to determine statistical significance. 

### 2.2. Quantitative Real-Time PCR

qRT-PCR analysis was used to evaluate the expression of RASSF1A in primary ovarian tumors essentially as described previously [12] using the following primers to RASSF1A: forward, 5′-GGACGAGCCTGTGGAGTG-3′, and reverse, 5′- TGATGAAGCCTGTGTAAGAACC-3′. *β*-actin was used as the reference gene. Sequences of the *β*-actin primers have been previously described [13].

### 2.3. Western Blotting

Cells were lysed in modified RIPA buffer as described previously [14], and subjected to Western analysis using an RASSF1A polyclonal antibody described previously [6]. Tubulin antibodies were purchased from Santa Cruz biochemical (Santa Cruz, CA, USA). Protein concentrations in lysates were measured prior to loading using the Bio-Rad Protein Assay (Bio-Rad, Hercules, CA, USA). Densitometry was performed using a Densitometer and Quantity One software. Values are expressed as adjusted volume Optical Density units/mm^2^.

### 2.4. Caspase Assays

 Cells were plated in 12-well plates at 30% confluency and treated with Taxol the next day. 22 hours later cells were lysed and assays with the Caspase-Glo kit (Promega, Madison, WI, USA) as described by the manufacturer.

## 3. Results

### 3.1. RASSF1A Downregulation Correlates with Acquisition of Taxol Resistance in Primary Ovarian Tumors

**Figure 1:**
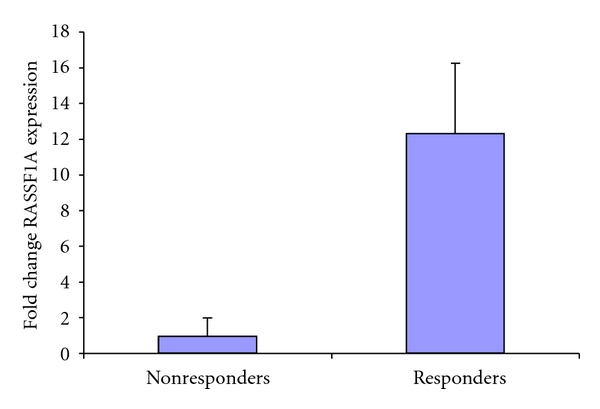
RASSF1A downregulation correlates with acquisition of Taxol resistance in primary ovarian tumors: qRT-PCR analysis of primary ovarian tumors correlates loss of RASSF1A expression with the development of Taxol resistance. Left column is relative expression of RASSF1A in Taxol-resistant patients; right column is relative expression in Taxol-sensitive patients. Data is expressed as fold change relative to the nonresponder group after normalization to *β*-actin expression. *t*-test was used to determine *P* was <.05.

mRNA isolated from the tumors of patients with stage III or IV papillary serous ovarian cancer [12] whose tumors were either responsive or nonresponsive to Taxol were assayed by qRT-PCR for the levels of RASSF1A expression. Ten samples were used for each group and the data expressed as fold change relative to RASSF1A expression in the nonresponder group, after normalization to the expression of *β*-actin. Those tumors which responded to Taxol showed considerably higher levels of RASSF1A mRNA than those which were resistant (Figure 1).

### 3.2. RASSF1A Knockdown Induces Resistance to Taxol

**Figure 2:**
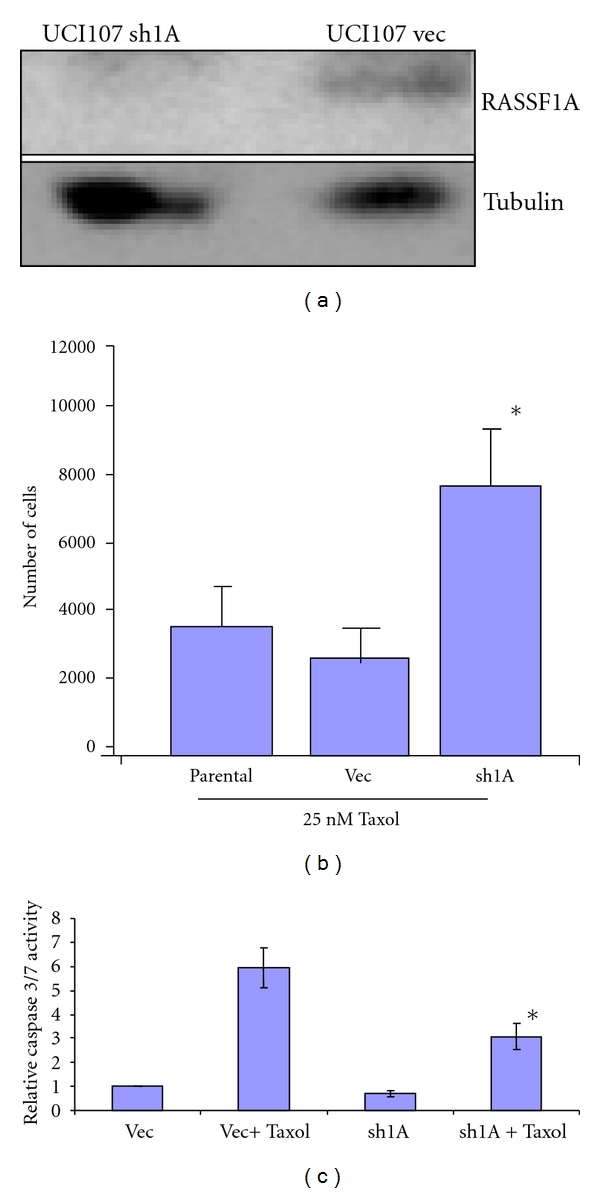
Loss of RASSF1A confers resistance to taxol-mediated apoptosis. A matched pair of RASSF1A ± cells was generated by stably knocking down RASSF1A expression in UCI-107 ovarian cancer cells using a RASSF1A-specific shRNA. Knockdown of RASSF1A was confirmed by western blotting. Tubulin served as a loading control (a). The UCI-107 RASSF1A ± cells were grown to 50% confluency and then treated with 25 nM Taxol or vehicle control 48 hours and cell number determined (b). Data represent an average of triplicate experiments, *P < 0.1 compared to parental or vector control cells. (c). The RASSF1A ± UCI-107 cells were treated with 25 nM Taxol for 22 hours and caspase activation measured as a readout for apoptosis using a luminescent caspase activation assay. Data represent the average of two assays performed in triplicate. *, statistically different from vector control cells treated with taxol, *P* < 0.05.

UCI-107 cells are a Taxol-sensitive ovarian cancer cell line [15]. We transfected the cells with our validated RASSF1A shRNA [9] or the empty vector and generated a stable matched pair by selection in puromycin. The cells were then western blotted for RASSF1A using our polyclonal rabbit antibody [6].  Figure 2(a) shows that RASSF1A expression was effectively knocked down in the shRNA transfected cell line.

The matched pair system was then challenged with Taxol for 48 hours and cell survival measured. Loss of RASSF1A enhanced the survival of the treated cells (Figure 2(b)). RASSF1A is a proapoptotic protein and loss of RASSF1A expression may induce resistance to apoptosis [9]. To determine if that may be the case in ovarian cancer cells treated with Taxol, we then examined the effects of RASSF1A expression on apoptosis after Taxol treatment. The RASSF1A ± UCI-107 cells were treated with Taxol for 22 hours and then assayed for apoptosis using the Promega Caspase 3/7 kit, which is a fluorescent measure of caspase activation.  Figure 2(c) shows that downregulation of RASSF1A promotes resistance to apoptosis induced by Taxol. We also observed a very slight reduction in the basal levels of caspase activation in the cells transfected with the RASSF1A shRNA.

### 3.3. Loss of RASSF1A Reduces the Ability of Taxol to Promote Microtubule Polymerization

RASSF1A binds microtubules and promotes their stabilization/polymerization [6, 7, 16]. Indeed, the effects of overexpressing RASSF1A in cells on tubulin is reminiscent of the effects of treating them with Taxol [6]. Moreover, downregulation of RASSF1A makes cells more sensitive to Nocodazole, a microtubule destabilizing drug [7]. Thus, we hypothesized that the presence of RASSF1A may be important to the ability of Taxol to induce microtubule polymerization. This would confirm RASSF1A loss as a component of the development of Taxol resistance in ovarian cancer and explain the results obtained in Figure 1.

**Figure 3:**
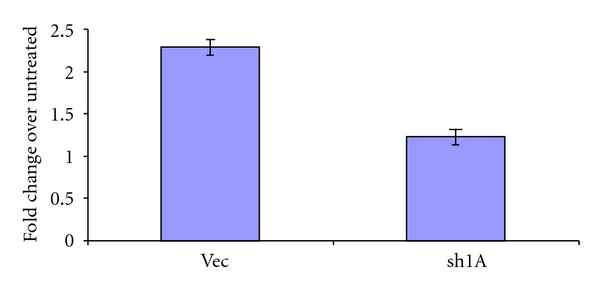
The ability of Taxol to promote tubulin acetylation is dependent on RASSF1A. The UCI-107 RASSF1A ± matched pair was treated with Taxol for 48 hours, cell lysates prepared and equal amounts of protein subjected to western blotting using antibodies specific for total or acetylated tubulin. The relevant bands from the western blot were quantified and average data from three experiments expressed as a ratio of acetylated tubulin to total tubulin to give a fold change. Knockdown of RASSF1A resulted in an approximately 50% reduction in the relative acetylation of tubulin, *P* = 0.042275.

 When Taxol polymerizes, it becomes acetylated and this has been used as a marker for polymerization [17]. The UCI-107 RASSF1A ± matched pair of cell lines was treated with Taxol. After 48 hours the cells were lysed and equal quantities of protein subjected to Western analysis first for total tubulin and then for acetylated tubulin using an acetylated tubulin specific antibody. The ratio of acetylated tubulin to total tubulin was determined by densitometric scanning of the western blots to permit quantitative assessment of the effects of the presence of RASSF1A.  Figure 3 shows that loss of RASSF1A expression reduces the ability of Taxol to promote microtubule polymerization.

### 3.4. Synergistic Restoration of RASSF1A Expression with DNMT Inhibitors

**Figure 4:**
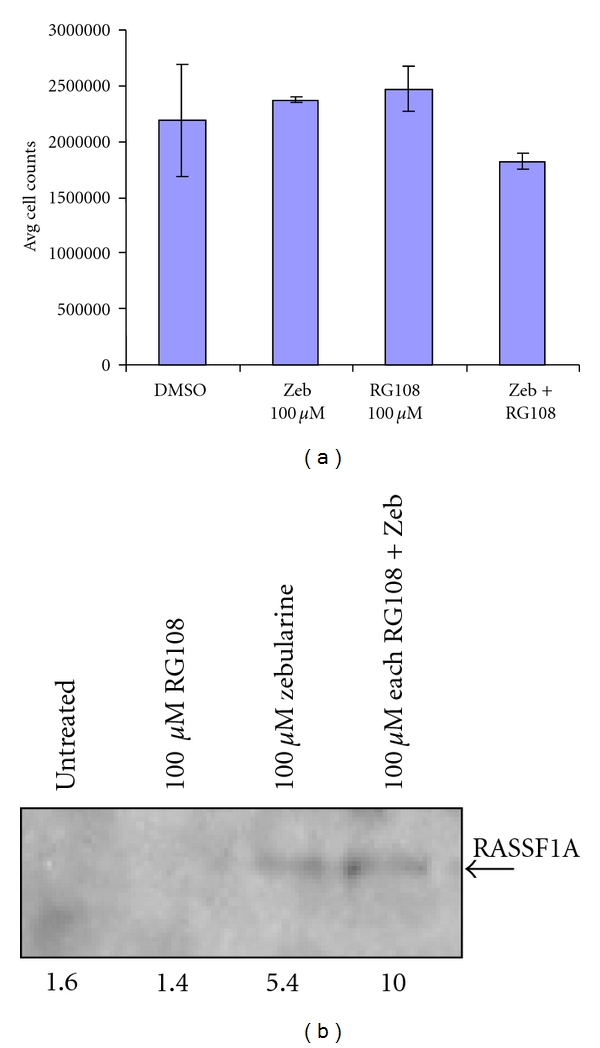
Synergistic reactivation of RASSF1A expression by RG108 and Zebularine. (a). RASSF1A negative A547 ovarian cancer cells were treated with DMSO, Zebularine, RG108 or Zebularine and RG108 in combination for 48 hours and surviving cells counted as a measure of toxicity. Treatment with either of the demethylating agents resulted in no significant difference in cell number. (b). A547 cells were treated with the indicated doses of RG108 and Zebularine alone or in combination for 48 hours and cell lysates prepared. Equal amounts of proteins were immunoprecipitated with an anti-RASSF1A antibody and the immunoprecipitates subjected to Western analysis for RASSF1A. Densitometric quantification of the bands is shown below the figure.

To examine the possibility that small molecule-induced restoration of RASSF1A expression might affect the cellular response to Taxol, we used the ovarian cancer cell line A547 that is negative for RASSF1A expression and exposed it to treatment with the DNA Methyl Transferase (DNMT) inhibitors Zebularine [10] and RG108 [11]. Zebularine has previously been shown to be active in restoring RASSF1A expression but is more specific and hence less toxic than the first generation DNMT inhibitor 5-AzaC [11, 18]. RG108 is a novel DNMT inhibitor that was designed to specifically inhibit the enzyme DNMT1 [19]. We also used the two in combination. Examination of the toxicity of RG108 and Zebularine allowed the determination of the minimal dose that provoked no detectable changes in cell growth or morphology. Combination of these two doses also resulted in no overt cell death (Figure 4(a)). Western analysis showed that Zebularine was more effective than RG108 at restoring RASSF1A expression but in combination their effects were greater than additive (Figure 4(b)).

### 3.5. Combined Epigenetic Therapy Restores Taxol Sensitivity

**Figure 5:**
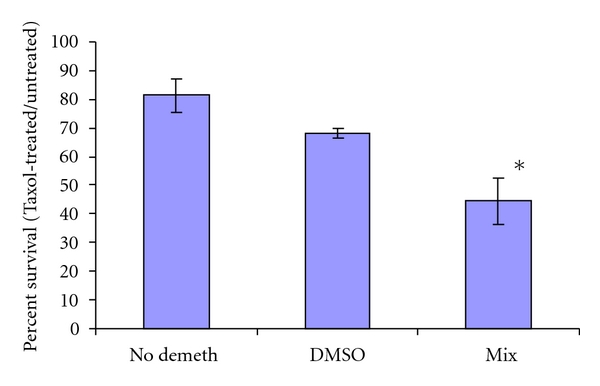
Synergistic epigenetic therapy enhances the taxol response of ovarian tumor cells. A547 cells were treated with carrier (DMSO) or a combination of RG108 and Zebularine (mix) for 48 hours, after which 400 nM Taxol was added and the cells incubated for an additional 48 hours. The number of viable cells was determined by trypan blue staining. Data are expressed as percent surviving cells relative to non-Taxol-treated cells for each condition.

Having determined that RG108 and Zebularine could act synergistically to restore RASSF1A expression at doses that were too low to induce cell toxicity, we examined the effect of the treatment on the Taxol response of the cells.  Figure 5 shows that A547 cells pretreated with the Zebularine/RG108 epigenetic therapy regimen exhibited an enhanced sensitivity to Taxol.

## 4. Discussion

 The RASSF1A tumor suppressor is frequently inactivated by an epigenetic process of aberrant promoter methylation in ovarian cancer [1]. RASSF1A complexes with microtubules and enhances their polymerization. Inactivation of RASSF1A results in an increased sensitivity to microtubule destabilizing drugs. Overall, the data suggests that RASSF1A plays an important role in the stabilization of microtubules. As the drug Taxol is thought to work in large part by stabilizing microtubules, we hypothesized that loss of RASSF1A expression might play a role in the development of resistance to Taxol. Our analysis of primary ovarian tumors showed that RASSF1A levels were much lower on average in Taxol resistant tumors. Based on this supporting evidence we proceeded to generate a matched pair of ovarian tumor cell lines that were identical other than for RASSF1A expression. Using this system, we showed that loss of RASSF1A expression caused a significant increase in the resistance of the cells to growth inhibition and apoptosis induction by Taxol.

 These data supported the idea that if we could restore RASSF1A expression then we might be able to restore Taxol sensitivity to a tumor cell. Using a combination of demethylating drugs we were able to restore RASSF1A expression. These drugs, RG108 and Zebularine, appear much less toxic than the established demethylating drug 5-Aza-C, even when used in combination (unpublished observation, G. Clark). The cells with restored RASSF1A expression proved much more sensitive to Taxol. Thus, we provide proof of principle for the use of epigenetic therapy to overcome Taxol resistance in ovarian cancer. Moreover, the methylation of the RASSF1A promoter might serve as a predictive marker for the effectiveness of Taxol based therapy.

 These studies focused on the role of RASSF1A in the Taxol response because of the apparent role of RASSF1A in supporting microtubule polymerization. However, RASSF1A has a general role in apoptosis and has now been shown to play a role in DNA repair. Thus, RASSF1A restoration might also be expected to enhance the effects of drugs which act by inducing apoptosis and DNA damage. Indeed, Zebularine has been shown to enhance the effects of Cisplatin in ovarian cancer models [20].

In these studies, we used Zebularine and RG108 as DNMT inhibitors. As they have different mechanisms of action, we hypothesized that they might have a synergistic activity. This would appear to be the case. As better agents arise that are more specific, for example Nanaomycin [21], the effectiveness and practicality of this strategy is likely to increase.

 RASSF1A exhibits an SNP, which is present in excess of 20% of the Caucasian population. This SNP produces a variant protein where Alanine 133 is substituted for a serine. The A(133)S variant protein is defective for interacting with certain isoforms of tubulin [22] and is defective for binding the microtubule association protein MAP1a [23]. Mutations close to this SNP can impair the ability of RASSF1C to promote microtubule polymerization [6]. Thus, it may be interesting to determine if the presence of this SNP may also affect the response of an individual to Taxol treatment.
